# The Architecture of Circulating Immune Cells Is Dysregulated in People Living With HIV on Long Term Antiretroviral Treatment and Relates With Markers of the HIV-1 Reservoir, Cytomegalovirus, and Microbial Translocation

**DOI:** 10.3389/fimmu.2021.661990

**Published:** 2021-04-19

**Authors:** Lisa Van de Wijer, Wouter A. van der Heijden, Rob ter Horst, Martin Jaeger, Wim Trypsteen, Sofie Rutsaert, Bram van Cranenbroek, Esther van Rijssen, Irma Joosten, Leo Joosten, Linos Vandekerckhove, Till Schoofs, Jan van Lunzen, Mihai G. Netea, Hans J.P.M. Koenen, André J.A.M. van der Ven, Quirijn de Mast

**Affiliations:** ^1^ Department of Internal Medicine and Radboud Center for Infectious Diseases, Radboud University Medical Center, Nijmegen, Netherlands; ^2^ HIV Cure Research Center, Department of Internal Medicine and Paediatrics, Faculty of Medicine and Health Sciences, Ghent University and Ghent University Hospital, Ghent, Belgium; ^3^ Laboratory for Medical Immunology, Department of Laboratory Medicine, Radboud University Medical Center, Nijmegen, Netherlands; ^4^ ViiV Healthcare, Brentford, United Kingdom; ^5^ Department for Genomics & Immunoregulation, Life and Medical Sciences 12 Institute (LIMES), University of Bonn, Bonn, Germany

**Keywords:** HIV, Th17 & Tregs cells, CD4+/CD8+ lymphocytes, B cell, HIV reservoir, CMV, Interferon gama (IFN-γ), Natural killer cell (NK cells)

## Abstract

Long-term changes in the immune system of successfully treated people living with HIV (PLHIV) remain incompletely understood. In this study, we assessed 108 white blood cell (WBC) populations in a cohort of 211 PLHIV on stable antiretroviral therapy and in 56 HIV-uninfected controls using flow cytometry. We show that marked differences exist in T cell maturation and differentiation between PLHIV and HIV-uninfected controls: PLHIV had reduced percentages of CD4+ T cells and naïve T cells and increased percentages of CD8+ T cells, effector T cells, and T helper 17 (Th17) cells, together with increased Th17/regulatory T cell (Treg) ratios. PLHIV also exhibited altered B cell maturation with reduced percentages of memory B cells and increased numbers of plasmablasts. Determinants of the T and B cell composition in PLHIV included host factors (age, sex, and smoking), markers of the HIV reservoir, and CMV serostatus. Moreover, higher circulating Th17 percentages were associated with higher plasma concentrations of interleukin (IL) 6, soluble CD14, the gut homing chemokine CCL20, and intestinal fatty acid binding protein (IFABP). The changes in circulating lymphocytes translated into functional changes with reduced interferon (IFN)- γ responses of peripheral blood mononuclear cells to stimulation with *Candida albicans *and *Mycobacterium tuberculosis.* In conclusion, this comprehensive analysis confirms the importance of persistent abnormalities in the number and function of circulating immune cells in PLHIV on stable treatment.

## Introduction

Combination antiretroviral therapy (cART) has drastically curtailed morbidity and mortality in people living with HIV (PLHIV) (1). Still, PLHIV remain at an increased risk for pneumococcal infections, *Mycobacterium tuberculosis* (*M. tuberculosis*) reactivation and impaired vaccine responses (2–6). Moreover, HIV infection predisposes to non-infectious comorbidities, such as cardiovascular disease and non-AIDS-related cancer, which share an underlying pathophysiological pathway characterized by a persisting and inappropriate activation of innate and adaptive immune cells (7, 8). Together, these observations point towards a disbalance in the homeostasis of the immune system, characterized by immunodeficiency on the one hand, and chronic inflammation on the other hand.

HIV-1 preferentially infects and kills activated CD4+ T cells, leading to rapid and severe CD4+ T cell depletion in the gut and increased microbial translocation (9–12). A small proportion of these cells remains latently infected with replication competent virus and defective proviruses, called the HIV-1 reservoir (13). The HIV-1 reservoir and increased microbial translocation, together with lifestyle factors and co-infections such as cytomegalovirus (CMV) may all contribute to the disrupted immune function in PLHIV (10, 13–17). However, published data have shown inconsistent or even contradictory findings. Heterogeneity in study populations, limited sample sizes of study populations and differences in lifestyle factors, including use of tobacco and recreational drugs, may underlie these inconsistencies and emphasize the need for an integrative approach in evaluating the immune system in PLHIV on stable cART.

The Human Functional Genomics Project (HFGP) aims to disentangle variation in the immune system in different cohorts of healthy individuals and individuals with underlying conditions. It combines multiple levels of analyses and data integration, including demographic and lifestyle data, data from ‘omics technologies’, and functional immune data (18). As part of this project, we previously identified relevant environmental and host factors for circulating white blood cell (WBC) populations in healthy individuals (19). Embedded within the HFGP, we established a cohort of 211 virally suppressed PLHIV (200HIV) and showed that these individuals exhibited a sustained pro-inflammatory immune phenotype with priming of the interleukin (IL)-1β pathway (20). In the present study, we used the same cohort to comprehensively assess the peripheral WBC composition during treated HIV infection with a special focus on the adaptive immune system. We compared their WBC populations with those of healthy individuals and assessed associations with demographic and lifestyle factors, different HIV-specific factors, and *ex vivo* cytokine responses of peripheral blood mononuclear cells (PBMCs) to stimulation with different bacterial, fungal and viral antigens.

## Methods

### Study Population

This study is part of the HFGP (www.humanfunctionalgenomics.org) (18). Between 14 December 2015 and 6 February 2017, individuals living with HIV were recruited from the HIV clinic of Radboud university medical center. Inclusion criteria were Caucasian ethnicity, age ≥ 18 years, receiving cART > 6 months, and latest HIV-RNA levels ≤200 copies/ml. Exclusion criteria were: signs of acute or opportunistic infections, antibiotic use <1 month prior to study visit, and active hepatitis B/C. The control population consisted of 56 healthy adult individuals (56P cohort), who did not suffer from any acute or chronic conditions and who were longitudinally sampled four times in three-month intervals. Inclusion, sampling and sample processing of both cohorts were conducted simultaneously and by the same personnel. The 56P participants were selected as a subset of a larger cohort of 534 healthy individuals (500FG) which was phenotypically assessed two years earlier according to the same methods (19, 21). Differences in cell-cell associations were compared between 200 HIV and 500FG cohort. The reason is that the larger sample size of this control cohort (n=534 vs n=56) improved statistical power, whilst batch effects between PLHIV and 500FG were deemed of less significance when comparing within-group correlations between cohorts. General information from all participants was recorded in the Castor Electronic Data Capture program (Castor EDC, CIWIT B.V., Amsterdam, The Netherlands), using questionnaires on socio-demographic information, lifestyle and life-events. Clinical data were extracted from medical files and the ‘Stichting HIV Monitoring’ registry (Amsterdam, The Netherlands).

### Ethics

The study protocols were approved by the Medical Ethical Review Committee region Arnhem-Nijmegen (ref. 42561.091.122) and conducted in accordance with the principles of the Declaration of Helsinki. All study participants provided written informed consent.

### Cell Processing

Venous blood was collected in sterile 10 ml EDTA BD Vacutainer^®^ tubes between 8 and 11 am and processed within 1-4 hours. Cell counts were determined using a Sysmex XN-450 automated hematology analyzer (Sysmex Corporation, Kobe, Japan). Erythrocyte-lysed whole blood samples were obtained after 10 minutes incubation of 1.5 ml EDTA-anticoagulated blood with lysis buffer containing 3.0 M NH_4_Cl, 0.2 M KHCO_3_ and 2 mM Na_4_EDTA. The remaining WBC were washed twice, by adding 25 ml phosphate-buffered saline 1x (PBS, Braun, Melsungen, Germany) and centrifuging at 452 x g for 5 min at room temperature. Before staining, cells were resuspended in 300 μl of PBS enriched with 0.2% bovine serum albumin (BSA, Sigma-Aldrich, Zwijndrecht, Netherlands). Isolation of PBMCs was performed by density centrifugation of EDTA-anticoagulated blood diluted 1:1 in pyrogen-free PBS over Ficoll-Paque (VWR, Amsterdam, The Netherlands) as described previously (22). Cells were adjusted to 5.0 x 10^6^ cells/ml.

### Flow Cytometry

**Supplementary Table 1** summarizes the antibody clones and the fluorochrome conjugates used for the different panel fluorescent staining mixes. Staining was performed on 100 μl/well erythrocyte-lysed blood (panel 1-3,5) or 0.5 x 10^6^ cells/well freshly isolated PBMCs (panel 4). Cells were stained according to previously described procedures (see also **Supplementary Methods**) (19).

Samples were measured on a 10-color Navios flow cytometer (Beckman Coulter, Fullerton, CA, USA), equipped with three solid-state lasers (488 nm, 638 nm, and 405 nm) (19). Gating was conducted manually and verified by two independent specialists to prevent gating errors. Samples were analyzed within 4-5 hr after blood collection, using five distinct and complementary 10-color antibody panels: 1. general; 2. T cell; 3. B cell; 4. intracellular T cell/Treg; 5. chemokine receptors (CCR). Staining and gating strategies can be found in **Supplementary Figure 1**. Flow cytometry data were analyzed using Kaluza software version 1.3. In our analyses, we focused on a set of 108 manually annotated WBC subsets based on the original 500FG study (panel 1-4) (19), with the addition of a fifth panel in which we classified monocytes, CD4+ memory and regulatory T cells, and CD8+ cells according to their expression of the CXCR3, CCR4, and CCR6 (panel 5).

### PBMC Stimulation Experiments

Freshly isolated PBMCs were incubated with different stimuli (**Supplementary Table 2**) including bacterial (*Staphylococcus aureus*, *M. tuberculosis*, *Streptococcus pneumoniae [S. pneumoniae]*), fungal (*Cryptococcus gattii*, C*andida albicans [C. albicans]* hyphae and yeast) and other relevant antigens (Imiquimod, TLR7 ligand), in round-bottom 96-well plates (Greiner Bio-One, Frickenhausen, Germany) with 0.5 x 10^6^ cells/well at 37°C and 5% CO_2_ in the presence of 10% human pooled serum for seven days. Supernatants were stored at -20°C. Levels of the lymphocyte-derived cytokines IL-17, IL-22, and interferon (IFN)-γ were measured in the supernatants (PeliKine Compact or Duoset ELISA, R&D Systems).

### Plasma Markers

Serum levels of immunoglobulin (Ig)M and IgG were measured by immunonephelometry using a Beckman Coulter Imager and Beckman Coulter reagents. Measurements were standardized using certified European reference material 470 (ERM^®^-DA470). CMV IgG antibodies were measured in serum using ELISA (Genway Biotech Inc., San Diego, CA, USA) according to the manufacturer’s instructions. Markers of systemic inflammation, high-sensitive C-reactive protein (hsCRP) and soluble CD14 (sCD14), and microbial translocation, intestinal fatty acid binding protein (IFABP), were measured by ELISA (Quantikine, R&D Systems) according to the manufacturer’s instructions. IL-6 was measured using a SimplePlex Cartridge (Protein Simple, San Jose, CA, USA). Circulating plasma CCL20, IL-17A, and IFN-γ were measured using the commercially available Olink Proteomics AB Inflammation Panel as described previously (23, 24).

### Cell-Associated HIV-1 DNA and Cell-Associated HIV-1 RNA Quantification in CD4+ T Cells

The HIV reservoir was assessed by analyzing CD4+ cell-associated HIV-1 DNA (CA-DNA) and CA-RNA. In virally suppressed patients, the CA-DNA roughly equals the integrated HIV-1 DNA, being replication competent or not (25), while CA-RNA is associated with recent HIV-1 transcriptional activity and serves as a proxy for the active proviral reservoir (26). CA-DNA and CA-RNA were measured in triplicate by droplet digital PCR (ddPCR – Bio-Rad) in CD4+ T cells isolated using EasySep Human CD4+ T Cell Isolation Kit (Stemcell technologies, Vancouver, Canada) as described previously (27). CA-DNA was extracted by the DNeasy Blood & Tissue kit (Qiagen, Hilden, Germany) according to the manufacturer’s protocol with the addition of 75µl elution buffer on the column heated at 56°C for 10 minutes. CA-RNA was extracted using the Innuprep RNA kit (Westburg, Leusden, The Netherlands) with 30µl elution buffer. Total RNA was reversely transcribed to cDNA by qScript cDNA SuperMix according to manufacturer’s protocol (Quantabio, Beverly, MA, USA). CA-DNA was normalized by measuring the reference gene RPP30 (**Supplementary Table 3** and **Supplementary Methods**) in duplicate by ddPCR and expressed per million CD4+ T cells. CA-RNA was normalized using three reference genes per patient, (B2M, ACTB and GADPH), which were measured with LightCycler 480 SYBR Green I Master mix. HIV-1 RNA copies were divided by the geometric mean of the reference genes and expressed per million PBMCs. ddPCR data analysis was performed using the ddpcRquant tool with standard settings for thresholding and absolute quantification (28).

### Statistical Analyses

A detailed description of the statistical methods can be found in the **Supplementary Methods**. Given the impact of cohort differences in the absolute cell numbers of main WBC types (e.g. CD4+) on the numbers of their subsets (e.g. CD4+ regulatory T cells [Tregs]), results were primarily reported as WBC percentages, unless stated otherwise. WBC percentages were calculated by dividing the cell count of each subpopulation by its respective parent (one level up) or, where relevant, grandparent (two levels up; **Supplementary Table 4**). Data curation of cytokine, soluble marker, and immunoglobulin data was done according to previous methods (21). For comparisons between cohorts, we used all four longitudinally collected data points from the 56P cohort as independent measurements. Because of the known seasonality effects on WBC, this approach was considered preferable over selecting one of the data collection points or summarizing the data points. Data were normalized using an inverse rank transformation algorithm.

Comparisons in baseline characteristics between groups were made using Student’s t-test (or Mann-Whitney U test) for continuous variables, and Pearson’s Chi-square test (or Fisher’s exact) for categorical variables. Linear regression was used to compare WBC between cohorts, and to calculate associations between WBC and clinical and virological factors. All analyses were corrected for age, sex, time since January 2015 and seasonal effects. Cytokine analyses between cohorts were also corrected for CD4+ and CD8+ T cell percentages. Spearman correlations were used as the distance metric for unsupervised hierarchical WBC count clustering. Two-sided FDR-corrected p-values < 0.05 were considered statistically significant (29). Effect sizes are reported as Spearman’s Rho or standardized beta coefficients (β). Data were analyzed using the statistical programming language R (version 3.4.3, R Core Team, 2012).

## Results

### General and HIV-Specific Characteristics

Data from 211 PLHIV and 56 controls were analyzed in this study (**Table 1**). PLHIV were older (median [IQR] 52.5 [46.2 – 59.4] vs 30.0 [25.1 – 52.2] years, *p<*0.0001) and more often male (193/211 [91.5%] vs 34/56 [60.7%], *p<*0.0001) than controls. PLHIV had received cART for a median of 6.6 (4.2–11.9) years and 205/210 (98%) had plasma HIV-1 RNA <50 copies/mL at time of study visit. Analyzing the HIV reservoir, we found detectable levels of total CA-DNA in 207/208 (99.5%) and CA-RNA in 210/210 (100%); CA-DNA and CA-RNA levels were highly correlated (**Supplementary Figure 2A**; R=0.68, p<2.2·10^-16^). In general, PLHIV with higher CA-DNA and CA-RNA levels were older, had been living with and treated for HIV for a longer period, and had lower nadir CD4+ T cell counts (**Supplementary Figure 2A**). We observed no significant differences in CA-RNA and CA-DNA between PLHIV with plasma HIV-1 RNA <50 copies/mL (205/210 [98%]) and those with plasma HIV-1 RNA 50-200 copies/mL (5/210 [2%]; **Supplementary Figure 2B**). PLHIV with at least once a viral load of >50 copies/mL during year prior to study visit (23/210 [11.0%]) had higher levels of CA-RNA (p=0.0077), but no differences in CA-DNA (p=0.076; **Supplementary Figure 2B**).

**Table 1:** General characteristics.

Characteristic	PLHIV (n = 211)	HC (n = 56)	P-value
Age, years	52.5 (46.2 – 59.4)	30.0 (25.1 – 52.2)	<0.0001
Sex, female, n/N (%)	19/211 (9.0)	22/56 (39.3)	<0.0001
BMI, kg/m^2^	24.2 (22.1 – 26.1)	23.6 (21.6 – 25.2)	0.41
Time since HIV diagnosis, years	8.5 (9.5)	–	–
Way of transmission, n/N (%)		–	–
MSM	159/211 (75.4)	–	–
Heterosexual contact	9 (4.3)	–	–
IDU	3 (1.4)	–	–
Other or unknown	40 (19.0)		
Nadir CD4^+^ cell count, cells/μl	250 (130 - 360)	–	–
CD4^+^ count, cells/μl	660 (480 - 810)	–	–
Zenith HIV-RNA, copies/mL	100 000 (50 000 - 391182)	–	–
HIV-RNA >50 copies/mL ≤1 yr. prior to inclusion, n/N (%)	23/210 (11.0)	–	–
CA-DNA (copies per million CD4+ cells)	1547 (584 - 2802)		
CA-RNA (copies per million CD4+ cells)	157 (74 - 299)		
cART-naive, n/N (%)	30/211 (14.2)	–	–
Time on cART, years	6.6 (4.2 – 11.9)	–	–
ARV classes, n/N (%)			
NNRTI	63/211 (29.9)	–	–
PI	32/211 (15.2)	–	–
INSTI	141/211 (66.8)	–	–
Co-medication, n/N (%)			
Cholesterol lowering drugs	58/211 (27.5)	–	–
Antihypertensive drugs	50/211 (23.7)	–	–
Metformin	9/211 (4.3)	–	–
Active smoking, n/N (%)	63/211 (29.9)	2/56 (3.6)	<0.0001
Heavy drinking, n/N (%)^*^	28/211 (13.3)	11/56 (19.6)	0.29
Regular substance use, n/N (%)*^†^*	61/211 (28.9)	3/56 (5.4)	<0.0001

Data depicted as median (IQR) unless stated otherwise. Data analyzed using Mann-Whitney U or χ^2^ (or Fisher’s exact) where applicable.

^*^Classified according to the CDC definition: for men, ≥15 drinks per week and for women, ≥8 drinks/week.

^†^Defined as use of any psychoactive substance (with the exception of alcohol and tobacco) during periods ≥ 1 time per week including ≥ 1 time during the 30 days prior to the study visit.

ARV, antiretroviral drug; BMI, body mass index; CA-DNA, CD4-cell-associated HIV-1 DNA; CA-RNA, CD4-cell-associated HIV-1 RNA; HC, healthy control; cART, combination antiretroviral therapy; INSTI, integrase inhibitor; IDU, intravenous drug use; MSM, men who have sex with men; NNRTI, non-nucleoside reverse transcriptase inhibitor; PLHIV, people living with HIV; PI, protease inhibitor.

### Alterations in WBC Composition and Cell-Cell Associations in Chronic HIV

**Figure 1:**
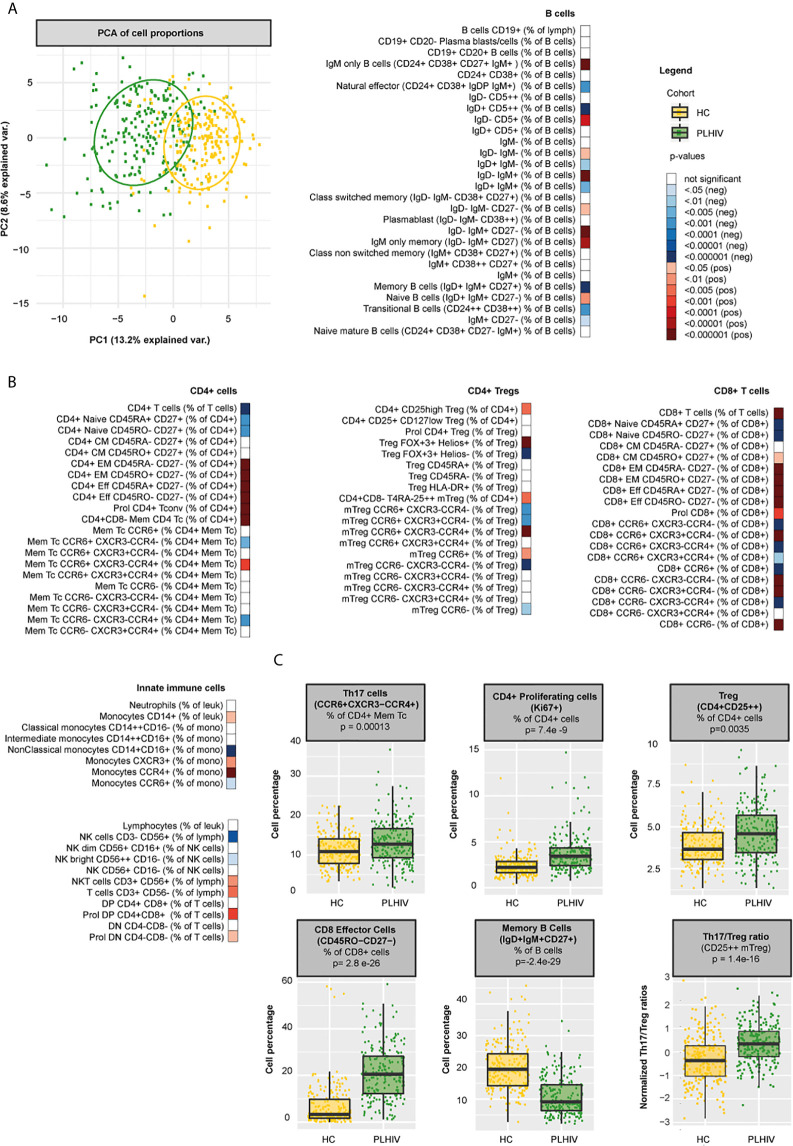
Differences in WBC percentages between PLHIV and healthy individuals **(A)** Principal component biplot showing standardized variance for the two principal component of all WBC subsets. The ovals represent the different cohorts. **(B)** Differences in WBC percentages (n=108 WBC subsets) between PLHIV (n=211) and healthy controls (n=56) **(C)** Boxplots showing examples of cohort differences in cell percentages and Th17/Treg ratios (Treg identification marker CD25++). Inverse-rank transformed data were analyzed using multiple linear regression and adjusted for age, sex, sampling time, and season. For color coding of the FDR-adjusted p-values see legend.

We analyzed 108 WBC populations, including 93/107 (87%) B and T cell subsets and 14/107 (13%) innate cell subsets (neutrophils, monocytes, natural killer [NK] and natural killer T cells [NKT]). **Figure 1** and **Supplementary Figure 3** show the differences in WBC composition between PLHIV and controls. An overview of median (IQR) WBC percentages and numbers in PLHIV and controls can be found in **Supplementary Table 5**.

Principal component analysis (PCA) of all WBC populations only explained a limited amount of the total variance. Still, the PCA plot showed clear differences in clustering between PLHIV and controls (**Figure 1A**). As expected, PLHIV exhibited an expansion of CD8+ and a reduction of CD4+ T cell numbers and percentages (**Figure 1B** and **Supplementary Table 5**). Functionally, CD4+ T cells comprise a diverse population of cells. CD4+ T helper (Th) cells fulfill essential roles for viral (Th1, CCR6-CXCR3+CCR4-), parasitic (Th2, CCR6-CXCR3-CCR4+), and mucosal (Th17, CCR6^+^CXCR3-CCR4+) immunity (30). In addition, CD4+ Tregs are essential for controlling inflammation (31). Despite reduced CD4+ T cell counts, numbers of all Th cell subsets (CD4+ CD45RA- CD25-) were markedly increased in PLHIV compared to controls (**Figure 1B** and **Supplementary Table 5**). Within the Th pool, Th2 percentages were reduced in PLHIV compared to controls, whereas Th1 percentages did not differ. Remarkably, Th17 percentages and numbers were increased in PLHIV (**Figures 1B, C** and **Supplementary Table 5**). While Treg percentages (of CD4+ cells), including highly suppressive Tregs co-expressing the transcription factors FoxP3 and Helios, were also increased in PLHIV, absolute Treg numbers were reduced (**Figures 1B, C** and **Supplementary Table 5**). This relative increase of Tregs may result from a loss of other CD4+ subsets (32). Among Tregs, we found no differences in the percentage of activated (HLA-DR+) and effector Tregs (CD45RA-). The relationship between pro-inflammatory Th17 cells and Treg must remain balanced to preserve functional immunity. Altered ratios have been described in untreated HIV (lower Th17/Treg), autoimmune disease and cancer (higher Th17/Treg) (9, 33, 34). Here, we found increased Th17/Treg ratios among virally suppressed PLHIV, irrespective of the Treg identification marker used (**Figure 1C** and **Supplementary Figure 4** **).** Furthermore, out of the Treg population, the percentage of Th17-like CCR6+ Tregs was increased in PLHIV, further contributing to a pro-inflammatory state. Developmentally, T cells evolve from naive T cells to antigen experienced central memory (CM), effector memory (EM), and effector cells (35, 36). HIV not only differentially affects functional subpopulations, but also disrupts these developmental stages. While naïve CD4^+^ and CD8^+^ T cells were reduced, memory and effector cells were expanded in PLHIV (**Figure 1B** and **Supplementary Table 5**). Likewise, we found higher percentages of proliferating (Ki67+) CD4+ and CD8+ T cells in PLHIV, indicating increased cell turnover. These results suggest a shift from naive cells towards terminally differentiated cells in HIV, even if viral replication is under control (37), which cannot be explained by age, sex, or season as we corrected our models for these factors.

**Figure 2:**
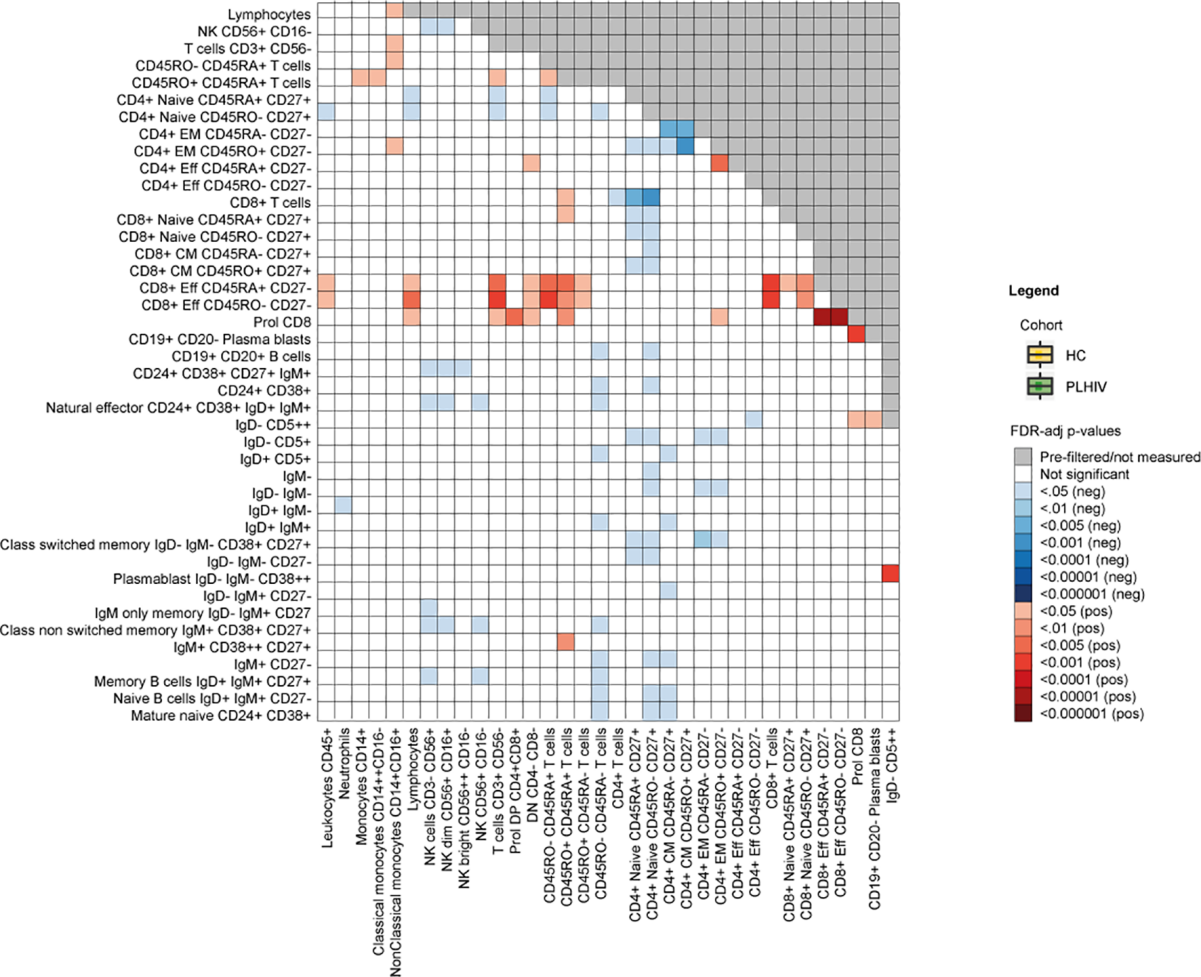
Differences in cell-cell associations between PLHIV and healthy individuals. Exploratory analysis depicting cell-cell associations between a total of 77 available WBC subsets that were significantly stronger (red) or weaker (blue) in PLHIV compared to healthy controls (n=534). FDR-adjusted p-values are obtained after 10 000 permutations and adjusted for age, sex, sampling time and season. HC, healthy control; PLHIV, people living with HIV; WBC, white blood cells.

Changes in the B cell compartment have also been described in PLHIV, including loss of CD27+ memory B cells (38, 39). Furthermore, viremia and low CD4+ T cell counts have been associated with the expansion of terminally differentiated B cells and immature B cells respectively (38, 39). In our study, we observed clear differences in B cell development in PLHIV illustrated by increased percentages of naïve B cells (IgD+IgM+CD27-) and reduced percentages of memory B cells (IgD+IgM+CD27+), transitional B cells (CD24++CD38++) and natural effector B cells (CD24+CD38+IgD+IgM+). In addition, the number of plasmablasts (IgD- IgM- CD38++) was increased in PLHIV, (**Figures 1B, C** and **Supplementary Table 5**). Adequate B cell maturation further requires optimal communication between B and T cells. We therefore sought to identify differences in WBC co-regulation between PLHIV and HIV-uninfected individuals by comparing cell-cell associations between PLHIV and participants from the 500FG cohort. Details of this 500FG cohort have been reported previously (500FG, n=534) (19). Using the same immunophenotyping techniques, we observed weaker correlations between naïve, CM and EM CD4+ T cells and several B cell subpopulations (including class-switch memory B cells) within PLHIV than in participants of the 500FG cohort, suggesting altered B/T cell interactions (**Figure 2**). These differences were not attributable to the influence of age, sex, or season as these factors were regressed out of the analysis.

Apart from changes in the adaptive cell compartment, we observed clear changes in the innate WBC compartment in PLHIV. Monocyte and NKT cell numbers and percentages were increased, whereas NK cell numbers and percentages were reduced, specifically the cytokine-producing NK bright cells (**Figure 1B** and **Supplementary Table 5**) Together, these data show a widespread functional and developmental dysregulation of the immune system in virally suppressed PLHIV. This dysregulation encompasses both the innate and the adaptive compartment and results in a pro-inflammatory immune environment with an expansion of monocytes, pro-inflammatory Th and effector T cells, and dysregulated B cell memory responses.

### Lifestyle, Demographic, and Clinical Factors Influence the WBC Composition During Chronic HIV Infection

**Figure 3:**
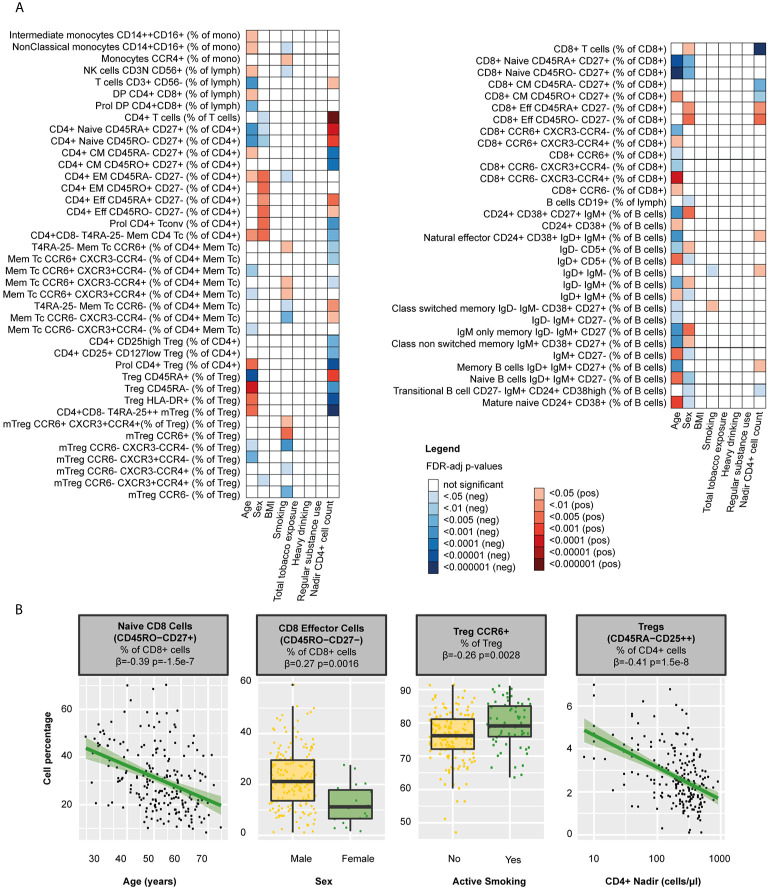
Clinical determinants of WBC percentages in PLHIV **(A)** Heatmap of WBC percentages (n=108 WBC subsets) that were significantly associated with any of the clinical determinants tested in 211 PLHIV; WBC subsets that showed no significant correlations with any of the parameters (n=37) have been removed from the figure. **(B)** Examples of WBC subsets that were significantly associated with age, sex, smoking, or CD4 nadir. Inverse-rank transformed data were analyzed using linear regression and adjusted for age, sex, sampling time, and season. For color coding of the FDR-adjusted p-values see legend. PLHIV, people living with HIV; WBC, white blood cells.

Apart from the effects of HIV, demographic and lifestyle-related factors may influence the composition of the circulating WBC populations. Indeed, we found that older age was associated with an increase of innate immune cells and differential effects on B and T cell populations, for example with lower percentages of naïve T cells (CD4+ T cells β=-0.29, p=0.00020; CD8+ T cells β=-0.29, p=0.00020) and B memory cells (β=-0.39, p=1.5·10^-7^) and higher percentages of memory T cells (e.g. CD8+ CM β=0.22, p=0.00064) and mature naïve B cells in PLHIV (β=0.25, p=0.00087, **Figure 3** and **Supplementary Table 6**). Sex-dependent influences of the WBC composition, reflected by more effector and EM cells and fewer naïve (B and T) cells in males in HIV infection, resembled those observed previously observed in healthy individuals (21, 40).

Lifestyle risk behaviors such as smoking [63/211 (29.9%)], heavy drinking [28/211 (13.3%)], and regular drug use [61/211 (28.9%)] were highly prevalent in PLHIV (**Table 1**). We found that packyears (reflecting total tobacco exposure) were associated with higher frequencies of neutrophils (β=0.22, p=0.033), Treg (β=0.22, p=0.033), CD8+ subsets (e.g. CCR6-CXCR3-CCR4+ β=0.24, p=0.025), and class switched memory B cells (β=0.20, p=0.042, **Supplementary Table 6**). Active smoking correlated with higher percentages of Th17 (β=0.21, p=0.031) and CCR6+ Tregs (β=0.26, p=0.0028, **Figure 3** and **Supplementary Table 6**). Neither heavy drinking, nor regular drug use affected the WBC composition.

We further assessed the effects of relevant clinical factors on the WBC composition in PLHIV, by exploring associations with the history of immune suppression and treatment-related factors. We found that nadir CD4+ cell counts were closely associated with both B and T cell percentages in PLHIV (**Figure 3** and **Supplementary Table 6**). For example, higher counts were associated with higher percentages of naïve CD4+ T cells (β=0.45, p=1.2·10^-9^) and memory B cells (β=0.18, p=0.034). In contrast, we observed no effects of the duration of HIV infection or cART (regimen) on the WBC composition (**Supplementary Tables 6**).

### HIV-1 Reservoir and CMV Affect the WBC Composition in Chronic HIV Infection

**Figure 4:**
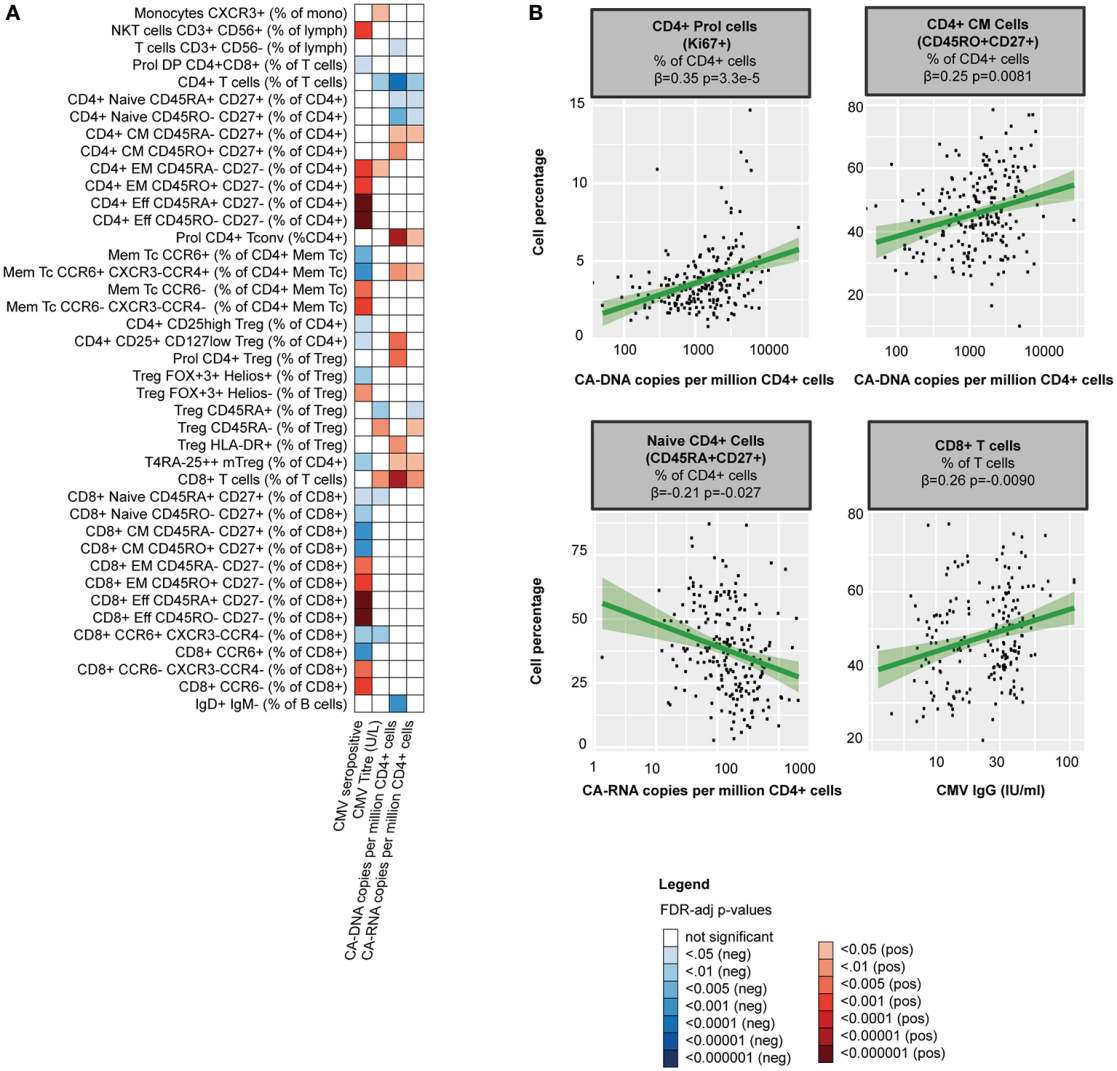
The HIV-1 reservoir, CMV serostatus and WBC percentages in PLHIV **(A)** Heatmap of significant associations between WBC percentages (total n=108), CMV co-infection (serostatus and IgG titers in 198 seropositive PLHIV) and markers of the HIV-1 reservoir (CA -DNA and CA- RNA) in 211 PLHIV; WBC subsets that showed no significant correlations with any of the parameters (n=41) have been removed from the figure. **(B)** Examples of WBC percentages that were significantly associated with the viral reservoir or CMV IgG titers. Inverse-rank transformed data were analyzed using linear regression and adjusted for age, sex, sampling time, and season. For color coding of the FDR-adjusted p-values see legend. CA-DNA, CD4-cell-associated HIV-1 DNA; CA-RNA, CD4-cell-associated HIV-1 RNA; CMV, cytomegalovirus; PLHIV, people living with HIV; WBC, white blood cells.

First, we explored the relationship with markers of the HIV reservoir. CA-DNA was negatively associated with CD4+ T cell percentages (β=-0.33, p=9.8·10^-5^) and positively with CD8+ T cell percentages (β=0.34, p=7.0·10^-5^). Within the CD4+ T cell pool, CA-DNA correlated with higher percentages of CM and Th17 cells (β=0.21, p=0.026 and β=0.24, p=0.0081 respectively; **Figure 4** and **Supplementary Table 7**) Higher CA-DNA was also associated with more CD4+ T cell proliferation (Ki67+, β=0.35, p=3.3·10^-5^) and Treg activation (HLA-DR+, β=0.23, p=0.0081). We found similar associations between T cells and CA-RNA, whereas we found no relation between T cells and the relative HIV transcription level (CA-RNA/CA-DNA ratios; **Supplementary Table 7**). Lastly, we observed several associations between CA-DNA and B cells: higher CA-DNA levels correlated with reduced percentages of IgD+ only memory B cells (β=-0.27, p=0.00068) and, albeit non-significantly, with reduced percentages of natural effector B cells (CD24+CD38+IgD+IgM+, β=-0.18, p=0.056), and class unswitched memory B cells (IgD+IgM+CD27+, β=-0.18, p=0.053). Exclusion of PLHIV with HIV-RNA 50-200 copies/mL at time of study visit (5/210 [2%]) did not change the main conclusions of our paper (**Supplementary Tables 8, 9**).

Second, CMV co-infection may contribute to immune dysregulation in both treated and untreated PLHIV. As CMV is known to affect WBC populations in healthy controls (41), we explored the association of CMV serostatus with WBC composition. 198 of the PLHIV (93.8%) were seropositive for CMV. In line with findings in healthy individuals (41), CMV seropositivity correlated with higher percentages of effector, EM T cells (e.g. with CD8+ effector cells β=0.43, p=1.7·10^-9^ and CD4+ EM cells β=0.30, p=0.00011; **Figure 4** and **Supplementary Table 7**) and NKT cells (β=0.29, p=0.00029), yet with lower percentages of Th17 cells (β=-0.26, p=0.00065). We observed no associations between B cell subsets and CMV.

### Microbial Translocation, Inflammation and Th17 Differentiation in Chronic HIV Infection

**Figure 5:**
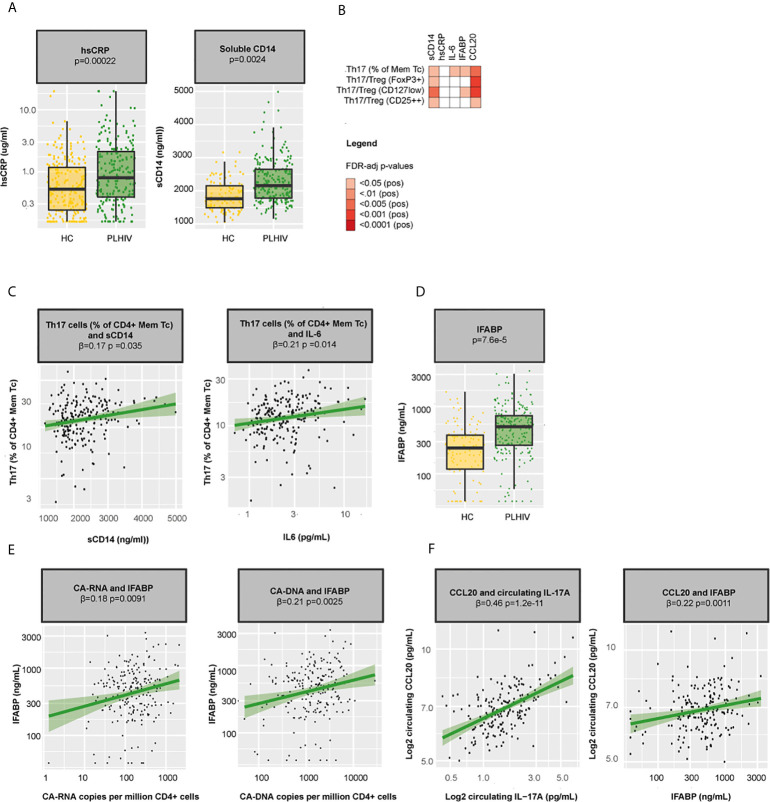
Inflammation, microbial translocation, and Th17 cells in PLHIV **(A)** Boxplots showing differences in hsCRP and sCD14 between PLHIV (n=211) and healthy controls (n=56). **(B)** Heatmap showing associations between Th17 percentages, Th17/Treg ratios and sCD14, hsCRP, IFABP, and CCL20. **(C)** Associations between Th17 percentages and sCD14 and circulating IL-6 in PLHIV. **(D)** Levels of the microbial translocation marker IFABP in PLHIV and healthy controls. **(E)** Associations between IFABP and CA-RNA and CA-DNA in PLHIV. **(F)** Associations between CCL20 and circulating IL-17A and IFABP. Inverse-rank transformed data were analyzed using linear regression analyses and corrected for sampling time. CA-DNA, CD4-cell-associated HIV-1 DNA; CA-RNA, CD4-cell-associated HIV-1 RNA; CCL20, Chemokine (C-C motif) ligand 20; HC, healthy control; hsCRP, high-sensitivity CRP; IFABP, intestinal fatty-acid binding protein; IL-6, interleukin 6; IL-17A, interleukin 17A; Mem Tc, CD4+ memory T cell; PLHIV, people living with HIV; sCD14, soluble CD14; Th17 percentages, T-helper 17 cell percentages (Mem Tc CCR6+ CXCR3-CCR4+ as percentage of CD4+ Mem Tc); Treg, regulatory T cells; WBC, white blood cells.

Our main findings include a loss of naïve T cells and an expansion of Th17 in PLHIV compared to healthy controls, which related with lower nadir CD4+ T cells counts and higher levels of CA-DNA. To further assess the potential underlying mechanisms, we explored the relationship between these WBC subsets, markers of chronic inflammation, and markers of microbial translocation. As discussed above, naïve T cells are able to differentiate into different Th subsets depending on the cytokine environment (42). We previously showed that PLHIV in our cohort exhibited a pro-inflammatory profile with increased levels of hsCRP (p=0.00022) and sCD14 (a marker of monocyte activation, p=0.0025; **Figure 5A**) as well as markedly elevated monocyte-derived cytokine responses, particularly IL-1β (20). Such a pro-inflammatory cytokine environment may push the differentiation of naive CD4+ cells into Th17 cells (42). Indeed, we observed positive associations between Th17 percentages and circulating IL-6 (β=0.21, p=0.014) and sCD14 (β =0.17, p=0.035; **Figures 5B, C**). Notably, Th17 cells fulfill an essential role in mucosal defense and gut Th17 cells are known to be severely depleted during acute HIV (9). To test whether gut integrity might have been compromised in our cohort, we measured levels of the microbial translocation marker IFABP and found increased levels in PLHIV compared to healthy controls (p=7.6·10^-5^; **Figure 5D**), suggesting ongoing microbial translocation in chronic treated HIV, especially in those with higher CA-RNA and CA-DNA (CA-RNA β =0.18, p=0.0091 and CA-DNA β=0.21, p=0.0025, **Figure 5E**). Higher levels of IFABP were associated with an expansion of peripheral blood Th17 (β=0.17, p0.043; **Figure 5B**). Homing of Th17 to the gut (and other tissues, such as the skin) is directed by the chemokine CCL20, which is produced by tissue and immune cells (neutrophils and monocytes) and binds uniquely to the CCR6+ receptor (43). Correspondingly, we found strong associations between CCL20 and Th17 percentages (β=0.22, p=0.0037), CCL20 and Th17/Treg (β=0.26, p=0.00081 for Th17/FoxP3+ Treg, β=0.27, p=0.00081 for Th17/CD127low Treg, and β=0.19, p=0.013 for Th17/CD25++ Treg), CCL20 and circulating IL-17A levels (β=0.47, p=1.2·10^-11^) and CCL20 and IFABP (β=0.24, p=0.0011; **Figure 5F**). Together, these results suggest that the pro-inflammatory environment in chronic HIV may promote the differentiation of circulating naïve CD4+ T cells into Th17 cells and that these changes may be associated with changes in gut permeability and gut homing.

### Functional Consequences of Changes in Adaptive Immune Cells in Chronic HIV

**Figure 6:**
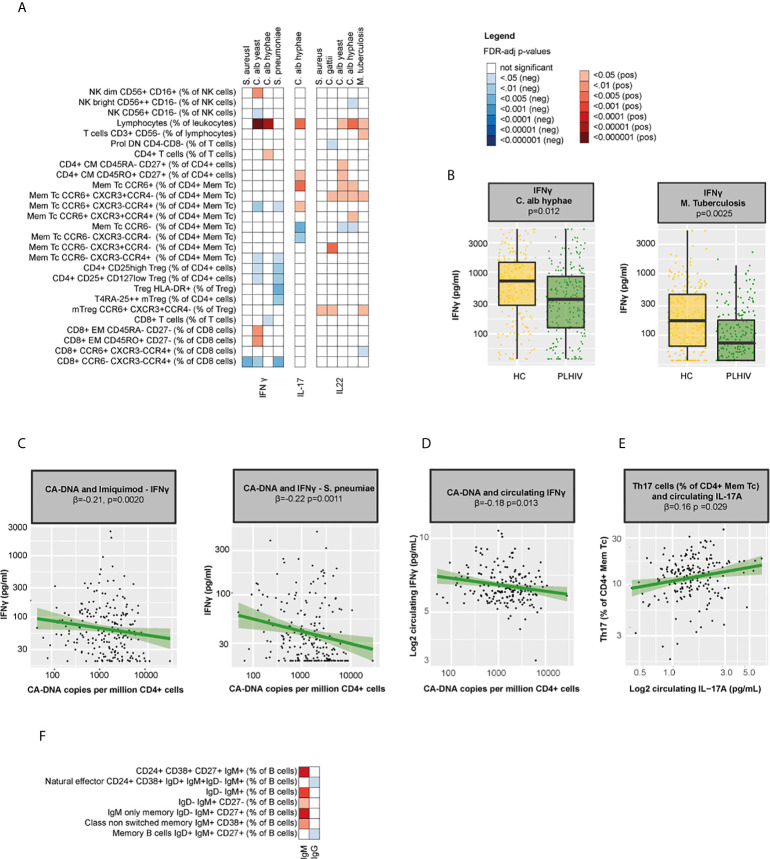
Functional consequences of WBC alterations in PLHIV **(A)** Heatmap of NK(T) and T cell percentages (total n=71) that were significantly associated with the ex vivo production of IFN-γ, IL-17, and IL-22 upon seven days ex vivo stimulation of PBMCs with different stimuli (n=7) in 211 PLHIV; WBC subsets that showed no significant correlations with any of the parameters (n=44) have been removed from the figure. **(B)** Boxplot showing differences in ex vivo IFN-γ responses upon stimulation with *C. albicans* and *M. tuberculosis* between PLHIV (n=211) and healthy controls (n=56). **(C)** Association between CA-DNA and *ex vivo* IFN-γ responses to stimulation with Imiquimod and S. pneumoniae in PLHIV. **(D)** Association between CA-DNA and circulating IFN-γ in PLHIV. **(E)** Association between Th17 percentages and circulating IL-17A in PLHIV. **(F)** Heatmap of B cell percentages (total n=28) that were significantly correlated with IgM or IgG levels in PLHIV; WBC subsets that showed no significant correlations with any of the parameters (n=21) have been removed from the figure. Inverse-rank transformed data were analyzed using linear regression analyses and corrected for sampling time. For cohort comparisons, data were corrected for age, sex, sampling time, season and the CD4+ and CD8+ cell percentages. For color coding of the FDR-adjusted p-values see legend. CA-DNA, CD4-cell-associated HIV-1 DNA; HC, healthy control; IFN-γ, interferon gamma; Ig, immunoglobulin; IL-17(A), interleukin 17(A); IL-22, interleukin 22; Mem Tc, CD4+ memory T cell; NK(T) cells, natural killer (T) cells; PBMC, peripheral blood mononuclear cell; PLHIV, people living with HIV; sCD14, soluble CD14; Th17 percentages, T-helper 17 cell percentages (Mem Tc CCR6+ CXCR3-CCR4+ as percentage of CD4+ Mem Tc); WBC, white blood cell.

As our results indicated significant changes in circulating immune cell populations in PLHIV, we next analyzed the possible functional consequences. First, we measured *ex vivo* cytokine responses of PBMCs after stimulation with different stimuli. We found strong correlations between NK and T-cell percentages and *ex vivo* IFN-γ, IL-17, and IL-22 responses (**Figure 6A** and **Supplementary Table 10**): IFN-γ responses correlated with percentages of NK dim, CD4+ and CD8+ EM cells, whereas IL-22 responses correlated with CCR6+ CM CD4+ cell percentages. As expected, Th17 percentages were associated with increased circulating IL-17A (β=0.16, p=0.029) and *ex vivo* IL-17 responses to *C. albicans* (β=0.19, p=0.047) and with reduced IFN-γ responses (**Figures 6A, E** and **Supplementary Table 10**). Despite the expansion of Th17 cells among PLHIV compared to controls, *ex vivo* responses of IL-17 or IL-22 did not differ, suggesting that the functional capacity of these cells may be compromised. In contrast, IFN-γ production upon stimulation with *C. albicans* hyphae (p=0.012) and *M. tuberculosis* (p=0.0025) was reduced in PLHIV (**Figure 6B**). Among PLHIV, lower *ex vivo* IFN-γ production to stimulation with Imiquimod (β =-0.21, p=0.0020) and *S. pneumoniae* (β=-0.22, p=0.0011) and lower circulating IFN-γ concentrations (β=-0.18, p=0.014) were associated with higher CA-DNA levels (**Figures 6C, D**). No associations were found between *ex vivo* cytokine production and CMV seropositivity.

Second, we measured serum immunoglobulins and found significant correlations between serum IgM and IgM+ B cell populations (**Figure 6F**), but no cohort differences in IgM levels [median (IQR) 0.76 (0.56 – 1.05) g/L in PLHIV versus 0.82 (0.65 – 1.21) g/L in controls, p= 0.22] or IgG levels [10.03 (8.34 - 11.59) g/L in PLHIV versus 9.10 (7.59 – 11.38) g/L in controls, p= 0.12]. These results indicate that while some of the alterations in adaptive immune function may be reversed by long-term cART (44, 45), others, such as impaired IFN-γ responses, remain.

## Discussion

In this study we show that, despite suppressive cART, the circulating innate and adaptive immune cell composition in PLHIV differs from that of HIV-uninfected individuals. We confirm that PLHIV exhibit a WBC profile characterized by proliferating memory and effector CD4+ and CD8+ T cells. While untreated HIV infection has been associated with a loss of circulating Th17 cells (9), we observed an expansion of circulating Th17 cells and increased Th17/Treg ratios during stable suppressive treatment, which was associated with plasma concentrations of IL-6, CCL20 and the microbial translocation marker IFABP. Furthermore, PLHIV showed clear changes in B cell maturation with reduced memory B cell percentages and increased plasmablast numbers. In the innate compartment, we observed an expansion of monocytes together with a loss of NK cells, specifically NK bright cells. In addition to age, sex, smoking, and CMV, we found strong associations between WBC populations and markers of the HIV-1 reservoir (CA-DNA and CA-RNA). Functionally, Th17 responses seemed to be preserved, whereas IFN-γ responses to *C. albicans* and *M. tuberculosis* were compromised, especially in those with higher CA-DNA. The compromised IFN-γ responses may affect host defense against some important bacterial pathogens (including *M. tuberculosis*) and the HIV-1 reservoir.

Prior studies have shown that untreated HIV infection results in a massive depletion of Th17 cells from the peripheral blood and the mucosa (9), a process that may partially be reversed by cART (46–48). We recently showed that PLHIV from the same cohort exhibited a pro-inflammatory profile with increased monocyte-derived cytokines, particularly IL-1β (20). IL-1β and IL-6 are among the critical cytokines driving differentiation of Th17 (42), and we postulate that these cytokines may have contributed to the higher circulating Th17 numbers. Th17 cells in the peripheral blood poorly reflect mucosal Th17 numbers (49) and it is possible that mucosal Th17 depletion with increased microbial translocation and altered Th17 recruitment occurs in the participants of our study. The gut-inflammatory marker generating islet-derived protein 3α (REG3α) would be of interest for future studies on the interplay between Th17 and epithelial gut damage in PLHIV (50).Concurrently to increased Th17 cells and pro-inflammatory Th17-like (CCR6+) mTregs, PLHIV showed increased peripheral blood Th17/Treg ratios. Increased Th17/Treg ratios have been linked to cardiovascular disease and atherosclerosis (33), cancer (34), and major depressive disorder (51), which are all highly prevalent in long-term treated PLHIV. Th17-mediated auto-immune diseases like psoriasis are also common among PLHIV, although they mostly occur during periods of severe immunosuppression and resolve upon cART initiation (52). Despite these changes in Th17, IL-17 and IL-22 cytokine responses did not differ between PLHIV and healthy controls. In contrast, we observed reduced *ex vivo* IFN-γ responses to *C. albicans* and *M. tuberculosis* in PLHIV. IFN-γ is predominantly produced by NK(T) cells, Th1, and CD8+ cells (44). Given that CD8+ cells were increased in PLHIV and Th1 cells did not differ between PLHIV and controls, we postulate that the reduced IFN-γ responses may have resulted from the loss of NK cells, which has been reported previously in both untreated and treated PLHIV (53, 54). In line with prior data, we observed an inverse relationship between IFN-γ responses and CA-DNA, suggesting that the failure to restore the NK cell compartment after cART initiation may be important for the containment of the HIV-1 reservoir (55).Moreover, IFN-γ is a key cytokine in the immune response against *M. tuberculosis* which remains an important pathogen in treated PLHIV (56). Improving IFN-γ responses, may therefore be relevant in the context of *M. tuberculosis* and HIV cure.

Different factors may contribute to the variation in T-cells repertoire in PLHIV. First, the effects of demographic factors such as age, sex, and smoking resembled those observed previously in healthy individuals (19, 41). Second, PLHIV in our study were almost universally coinfected with CMV and, in line with earlier data in HIV-infected and uninfected individuals, CMV seropositivity was associated with the expansion of effector and EM CD4+ and CD8+ T cells (14, 41, 57, 58). Of note, high CMV IgG levels may reflect frequent CMV reactivations or result from a stronger immune response (including adequate B/T cell interactions and B cell responses) and, consequently, fewer activations (59). Importantly, higher CMV IgG titers have been linked to microbial translocation and the development of non-AIDS-defining events such as cardiovascular disease (14, 60). Third, we found substantial associations between WBC subsets and CD4+ nadir and markers of the HIV-1 reservoir. Overall, CA-DNA showed more and stronger correlations with WBC subsets than did CA-RNA, which may be explained by the fact that CA-RNA levels, reflecting transcriptional activity, are low during viral suppression and subtle effects may be missed (13, 61).

Next to T cell dysfunction, HIV is characterized by aberrant B cell responses and B cell dysfunction. Using a different set of B cell markers than earlier studies in PLHIV (62, 63), we confirm their observations that percentages of naïve B cells are increased and memory B cells are reduced in PLHIV. Moreover, adequate B cell maturation requires optimal communication between T and B cells, which, according to our data, might be compromised in chronic stable PLHIV. B/T cell interactions take place in the germinal centers in lymph nodes and are orchestrated by follicular Th cells (Tfh) (64). As these cells are known to be highly permissive to HIV infection and serve as reservoirs during chronic infection, they could potentially explain these disrupted B/T cell interactions (64–66). Clinically, compromised B/T cells interactions may contribute to impaired immune responses to vaccination as well as increased risks for infections, such as invasive pneumococcal disease (5, 67). Finally, improvement of B/T cell communication is crucial for the development of broadly neutralizing antibodies and thus functional cure in PLHIV (68).

Our findings support the relevance of new immune modulating strategies in ART-treated PLHIV. Examples of interventions with potent anti-inflammatory properties in PLHIV include the IL-1β-inhibiting agent canakinumab (69) and the epigenetic modifier panobinostat (HDACi) (70, 71). Moreover, checkpoint inhibitors (e.g. those targeting PD1, PD-L1, and CTLA4) have been shown to, transiently, reverse latency of the viral reservoir, to restore cytotoxic T cell functions (72–75), and to enhance B-cell germinal responses to HIV-1 envelope vaccines (76). Further research is required to establish whether these agents are safe and effective long-term options to mitigate inflammation and to reverse immune exhaustion and HIV latency in stable HIV infection.

Several limitations should be considered when interpreting our data. First, the observational study design limits causal inferences. Second, generalizability of our findings to women, children and non-European populations requires further studying. Third, participants from the HIV cohort were older and more often male and age and sex both have known effects on the immune system (19, 21) To adjust for these differences, we used multivariable regression models which allowed us to take into account the effects of these independent predictors (age and sex) on our outcome of interest (HIV status). Fourth, we used a predefined set of markers and flow cytometry panels. While this standardized approach enhances validity and reproducibility and enabled us to compare our results with those of a large cohort of healthy controls, some interesting WBC subsets and markers for HIV have not been included (e.g. Tfh cells, CD38 HLA-DR co-expression, and PD-1/CD57 expression in the context of B/T cell interactions, T-cell activation, and immune cell exhaustion respectively). Furthermore, data on intracellular production of IL-17 and IFN-γ in maximally stimulated sorted cell populations (e.g. Th17 or NK cells) may provide a better understanding of the changes in cytokine production capacity per WBC subset. Finally, our WBC data are limited to the peripheral blood and we have no data on tissue-specific WBC composition (e.g. lymph nodes or the gut).

In summary, we show that the circulating innate and adaptive immune cell composition is altered in a large group of PLHIV receiving cART for more than six months. Furthermore, our findings suggest that some of the adaptive immune responses (Th17) are preserved while IFN-γ responses are compromised. Our comprehensive approach provides new insight into the changes in the immune cell architecture and functional immunity in treated HIV and highlights associations with the HIV-reservoir, underlining the need for early cART initiation. Our results are currently validated and extended in a multi-omics study including 2000 virally suppressed PLHIV (clinicaltrials.gov identifier: NCT03994835).

## Data Availability Statement

The raw data supporting the conclusions of this article will be made available by the authors, without undue reservation.

## Ethics Statement

The studies involving human participants were reviewed and approved by Medical Ethical Review Committee region Arnhem-Nijmegen (ref. 42561.091.122). The patients/participants provided their written informed consent to participate in this study.

## Author Contributions

WH, QM, MN, HK, IJ, and AV designed the study. WH, LW and MJ recruited and included the participants. WH, LW, MJ, WT, SR, BC, and ER performed the laboratory experiments. RH, LW and WH analyzed the data and interpreted the data together with QM, AV, MN, LK, HK, IJ and JL. LW, WH, AV and QM wrote the manuscript. All authors contributed to the article and approved the submitted version.

## Funding

QM, AV, and MN receive research support from ViiV Healthcare. The funders were not involved in the study design, data interpretation or the submission.

## Conflict of Interest

This study was supported by an AIDS-fonds (#P-29001) Netherlands and an ERC Advanced Grant (#833247). LV was supported by FWO (grant 1.8.020.09.N.00) and Collen-Francqui Research Professor Mandate. SR received a strategic basic research fund of the Research Foundation – Flanders (FWO, 1S32916N). TS and JL were employed by ViiV Healthcare.

The remaining authors declare that the research was conducted in the absence of any commercial or financial relationships that could be construed as a potential conflict of interest.
