# Supplementary material for: The Architecture of Circulating Immune Cells Is Dysregulated in People Living With HIV on Long Term Antiretroviral Treatment and Relates With Markers of the HIV-1 Reservoir, Cytomegalovirus, and Microbial Translocation
Source: Front Immunol. 2021 Apr 19;12:661990. doi: 10.3389/fimmu.2021.661990 (PMC8091964; doi:10.3389/fimmu.2021.661990)
Supplement: Supplementary file 1 [file DataSheet_1.docx]

**Supplementary Information**

**The Architecture of Circulating Immune Cells Is Dysregulated in People Living With HIV on Long Term Antiretroviral Treatment and Relates With Markers of the HIV-1**

**Reservoir, Cytomegalovirus, and Microbial Translocation**

**Authors**

Lisa Van de Wijer^1#^, Wouter A. van der Heijden^1#^, Rob ter Horst^1^, Martin Jaeger^1^, Wim Trypsteen^2^, Sofie Rutsaert^2^, Bram van Cranenbroek^3^, Esther van Rijssen^3^, Irma Joosten^3^, Leo Joosten^1^, Linos Vandekerckhove^2^, Till Schoofs^4^, Jan van Lunzen^4^, Mihai G. Netea^1,5^, Hans J.P.M. Koenen^3^, André J.A.M. van der Ven^1^, Quirijn de Mast^1^

*^1^ Department of Internal Medicine and Radboud Center for Infectious Diseases, Radboud university medical center, Nijmegen, The Netherlands*

*^2^ HIV Cure Research Center, Department of Internal Medicine and Paediatrics, Faculty of Medicine and Health Sciences, Ghent University and Ghent University Hospital, Ghent, Belgium*

*^3^ Department of Laboratory Medicine, Laboratory for Medical Immunology, Radboud university medical center, Nijmegen, The Netherlands*

*^4^ViiV Healthcare, Brentford, UK*

*^5^Department for Genomics & Immunoregulation, Life and Medical Sciences 12 Institute (LIMES), University of Bonn, Bonn, Germany*

*# The authors have contributed equally to the work and share first authorship*

******

***Supplementary Figure* 1 *Flow cytometry gating strategies of the general (A) and T cell (B) panels***

******

***Supplementary Figure* 1 *Flow cytometry gating strategies of the B cell(C) and Treg (D) panels***

******

***Supplementary Figure* 1 *Flow cytometry gating strategies of the chemokine receptor (CCR) panel (E)***

***
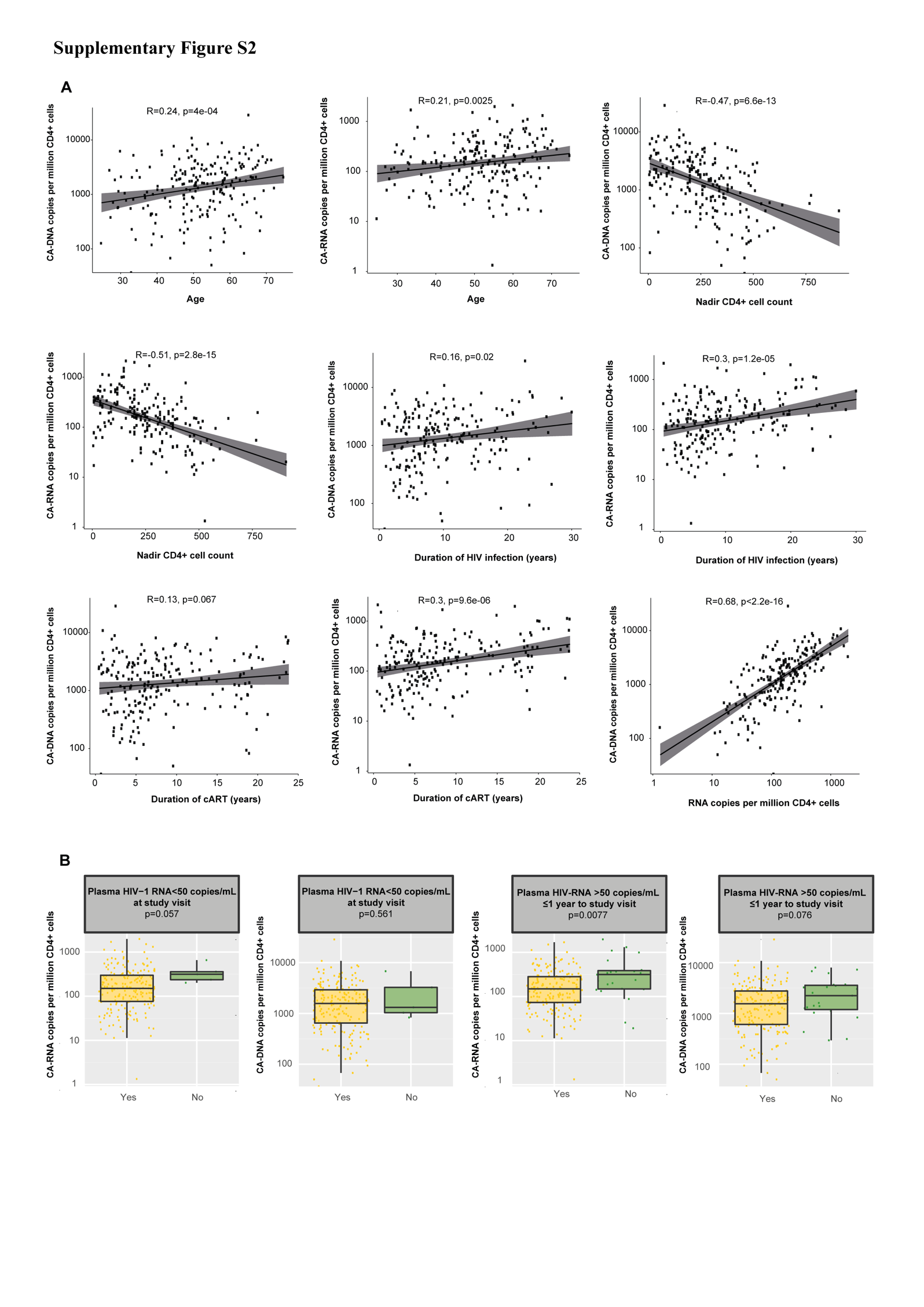
***

***Supplementary Figure* 2** *(A) Spearman’s correlations between CA-DNA and CA-RNA and clinical factors (B) Differences in CA-RNA and CA-RNA between PLHIV with (205/210 [98%]) and without HIV-1 RNA 50 <copies/mL (5/210 [2%]) at study visit and between PLHIV with (23/210 [11%]) and without plasma HIV-RNA>50 copies/mL (187/210 [89%]) in the year prior to study visit.*

*CD-DNA: CD4-cell associated HIV-1 DNA; CA-RNA CD4-cell associated HIV-1 RNA*

***Supplementary Figure* 3 *Differences in WBC percentages between PLHIV and healthy individuals (previous page)*** *Differences in WBC percentages between PLHIV (n=211) and healthy individuals (n=56) ordered in an hierarchical tree like structure in whole blood (A) and peripheral blood mononuclear cells ( B). Inverse-rank transformed data were analyzed using linear regression and adjusted for age, sex, sampling time, and season. For color coding of the FDR-adjusted p-values see legend.*

***
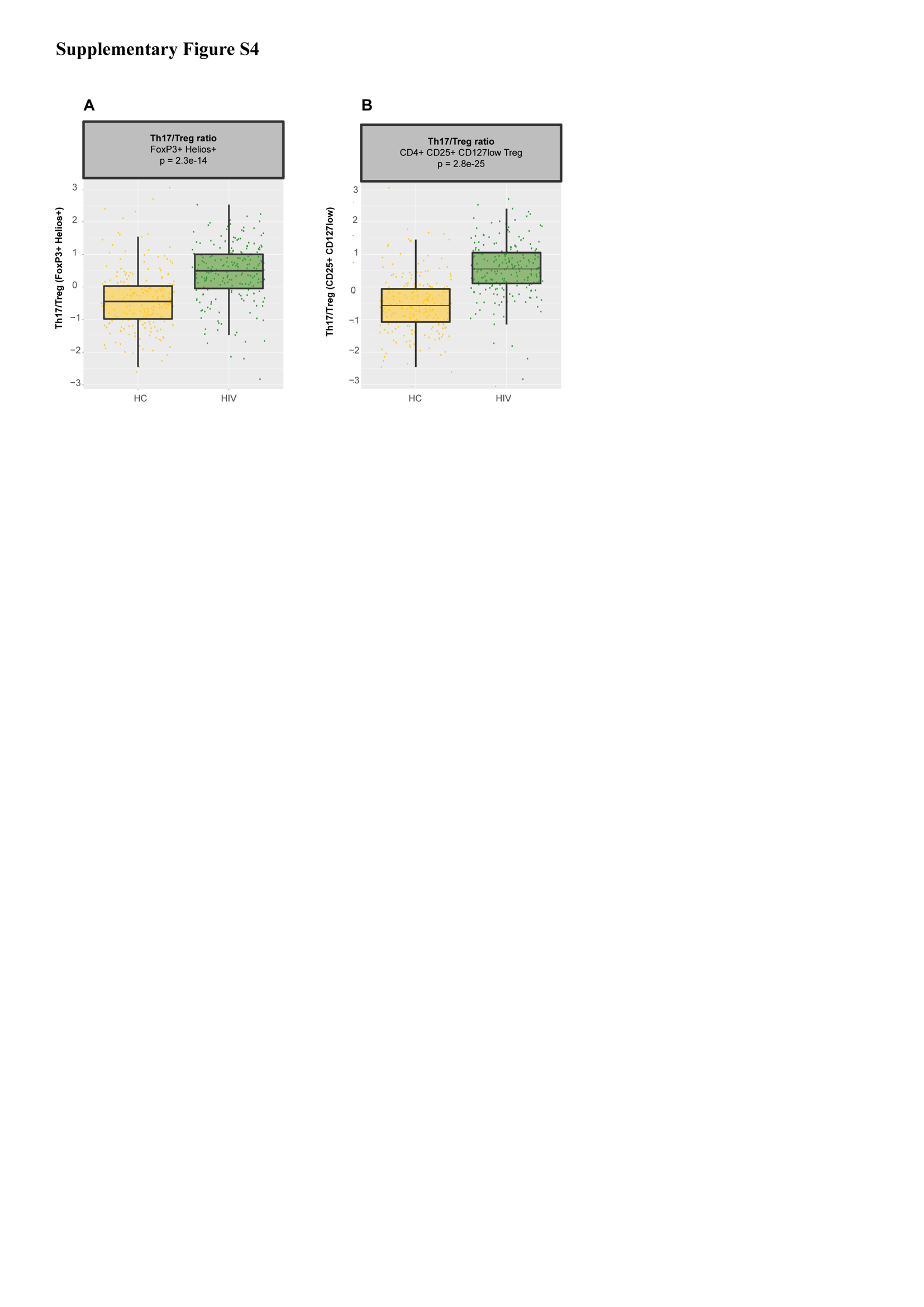
***

***Supplementary Figure 4 Differences in Th17/Treg ratios with different markers.*** *(A) Th17/Treg ratio based in which Tregs were identified using FoxP3 and Helios. (B) Th17/Treg ratio based in which Tregs were identified using CD25 and CD127. Results are comparable with results reported in Figure 1B*

*Th17: T-helper 17 (Mem Tc CCR6+ CXCR3-CCR4+); Treg: regulatory T cell*

**Supplementary Methods**

HIV-1 DNA and cell-assicioated HIV-1 RNA quantification in CD4+ T cells

See Methods for the description of the HIV-1 DNA and RNA extraction. Before PCR amplification, 8.65µl of genomic DNA was restricted by EcoRI (Promega) in a total volume of 10µl restriction digest for a minimum of 1 hour at RT. Total HIV-1 DNA and HIV-1 RNA were measured by adding respectively 2µl and 4µl in triplicates to ddPCR mix containing 10µl 2x ddPCR Supermix for Probes, 500nM primers and 300 nM probe (Table X). DNA was amplified by PCR with an initial denaturation step of 10min at 95°C, followed by a denaturation step for 30 sec at 95°C, an annealing/elongation step for 1 min at 56°C for 40 cycles, and a final step of 10min at 98°C. Total HIV-1 DNA was normalized by measuring the reference gene RPP30 (Table X) in duplicate by ddPCR and expressed per million CD4+ T cells. Droplets were read by QX200 droplet reader (Bio-Rad) and automatic threshold setting was done using ddpcRquant software^34^. For HIV-1 RNA normalization, three reference genes per patient, B2M, ACTB and GAPDH (Supplementary Table S3) were measured with LightCycler 480 SYBR Green I Master mix. HIV-1 RNA copies were divided by the geometric mean of the reference genes and expressed per million CD4+ T cells.

Immunophenotyping

*Staining*

For surface staining, cells were incubated in 25 μl surface staining master mix for 20 min at RT and washed twice in PBS + 0.2% BSA. Before acquisition, whole blood-derived cells were resuspended in 100 μl PBS + 0.2% BSA. For intracellular staining, surface-stained PBMCs were fixed and permeabilized using Fixation/Permeabilization solution (eBioscience, Vienna, Austria) for 30 min at 4°C protected from light. After washing the cells twice in permeabilization buffer (eBioscience, Vienna, Austria), cells were stained with 25 μl intracellular staining master mix for 30 min at 4°C protected from light. After a second washing step with permeabilization buffer, cells were resuspended in 100 μl PBS + 0.2% BSA for acquisition.

*Flow cytometry*

For each panel, single cells within the leukocyte (CD45^+^) population were identified by plotting the forward scatter (FSC) against FSC time of flight (FSC TOF), followed by characterization of the major myeloid or lymphoid lineages ^8^. The absolute number of WBC per ml of blood determined by the Sysmex XN-450 hematology analyzer was used to calculate absolute numbers of leukocyte (CD45^+^) cell subsets as measured by flow cytometry.

Panel 1 (general) identified granulocytes, lymphocytes (both by FSC, side scatter [SSC]) and monocytes (CD14^+^). Lymphocytes were further characterized into T cells (CD3^+^CD56^-^), NK cells (CD3^-^CD56^+^), NKT cells (CD3^+^CD56^+^ ), and B cells (CD19^+^HLA^-^DR^+^). Cell subsets were determined for T cells (CD4 and CD8), NK cells (CD56 and CD16) and monocytes (CD14 and CD16). Panel 2 (T cell) covered CD4^+^ regulatory T cells (Treg; CD4^+^CD25^+^CD127 low) and maturation stages of CD4^+^ and CD8^+^ T cells (using CD45RA/CD27 and CD45RO/CD27). Panel 3 (B cell) explored CD19+ B cell maturation stages by the expression of IgM/IgD and/or CD24/CD38 and B cell subsets by differential CD19/CD20 and IgD/CD5 expression, as previously described ^8^. In panel 4 (intracellular T cell/Treg), the major T cell populations (CD4, CD8 and Treg) were identified and analyzed for proliferation status by intracellular Ki67 expression. Tregs (CD4^+^CD25^+^CD127low FoxP3^+^) were analyzed for expression of Helios, CD45RA, and HLA-DR. Absolute cell counts in this panel were calculated as described above by subtracting the granulocyte number (determined by panel 1) from the PBMC WBC counts (Sysmex XN-450). In panel 5 (chemokine), monocytes and T cell populations (CD8, CD4 including memory T cells [Mem Tc; CD45RA^-^CD25^-^] and Treg [CD45RA^-^CD25^++^]) were analyzed for the expression of different cc-chemokine receptors (CCR): CXCR3 (CD183), CCR4 (CD194), and CCR6 (CD196).

Statistical methods

*Linear regression analysis*

The following regression formula was used:

*Y1 ~ X1 + age + sex + sin(2 * pi * numDaysFromJan2015/365) + cos(2 * pi * numDaysFromJan2015/365) + numDaysFromJan2015*

where sin(2 * pi * numDaysFromJan2015/365) and cos(2 * pi * numDaysFromJan2015/365) capture seasonality patterns with a periodicity of one year^15^. The cell count data showed some slight drift over time which was corrected using the linear term *numDaysFromJan2015*, indicating after how many days after Jan 1st 2015 the sample was collected.

*Analysis of correlation structures between 200HIV and 500FG*

Correlations were performed on absolute WBC counts instead of WBC proportions.

To properly ascertain cell count correlations, we first corrected the IRT-normalized cell counts for age, sex, and seasonal effects, by regression out these factor using a model similar to the one described above. We calculated inter-cohort differences in strength of cell count associations using the following method. First, we defined the test statistic:

*D_real=correlation_X1_Y1_cohort1 - correlation_X1_Y1_cohort2=C1-C2*

With hypotheses: *H0: c1=c2 and H1: c1≠c2*

where X1 and Y1 refer to the different cell types. Within each cohort the ID-labels were shuffled 10,000 times for each WBC type and the correlation between the cell types in both cohorts were calculated for all 10,000 permutations, yielding 10,000 unique correlation differences.

*D_permuted = correlation_X1_Y1_cohort1 - correlation_X1_Y1_cohort2*

P-values were obtained using two different methods. First, P-values were calculated by dividing the number of times the absolute shuffled test statistics were higher than the real absolute test statistic.

*(number of times abs(D_permuted) >= abs(D_real)) + 1)/number of permutations*

Second, p-values were obtained by applying a half-normal distribution to the absolute D_permuted values. P-values were calculated out of the percentage of the area with absolute permuted values higher than the real correlation. This second approach yielded more precise p-values without increasing the number of permutations (as for the discrete permutation analysis the lowest possible p-value is *1/number of permutations)*. Here, the p-values from the fitted distribution are reported. Using the p-values from the discrete method (first method) showed very similar pattern, and therefore selecting one method over the other does not change the overall conclusions.
